# Urine Organic Acids as Metabolic Indicators for Global Developmental Delay/Intellectual Disability in Chinese Children

**DOI:** 10.3389/fmolb.2021.792319

**Published:** 2021-12-22

**Authors:** Baiyu Chen, Yalan Zhan, Miriam Kessi, Shimeng Chen, Juan Xiong, Xiaolu Deng, Lifen Yang, Jing Peng, Fei Yin, Fang He

**Affiliations:** ^1^ Department of Pediatrics, Xiangya Hospital, Central South University, Changsha, China; ^2^ Hunan Intellectual and Developmental Disabilities Research Center, Changsha, China

**Keywords:** intellectual disability, global developmental delay (GDD), urine organic acids, metabolites, children

## Abstract

**Objective:** The purpose of this study was to search for differential metabolites in urine organic acids, and to characterize metabolic features that can be used to identify metabolites for exploration of global developmental delay (GDD)/intellectual disability (ID) etiology and pathogenesis.

**Methods:** We screened positive test results that could explain GDD/ID from 1,253 cases, and the major differential metabolites in 132 urine organic acids from the 1,230 cases with negative results (863 GDD cases, 367 ID cases), and 100 typically developing children (TD). Non-supervisory principal component analysis and orthogonal partial least squares discriminant analysis were used to develop models to distinguish GDD/ID from TD children, and to detect major differential metabolites.

**Results:** We get 23 positive results that could identify the cause of GDD/ID from 1253 cases diagnosed with GDD/ID. Among 1,230 negative results, we get the differential metabolites of the GDD group and the ID group had the same trend compared with the TD group. Twenty four differential metabolites were obtained from the GDD group, and 25 from the ID group (VIP > 1.0, *p* < 0.01). These differential metabolites were mainly related to the following pathways: the synthesis and degradation of ketone bodies, citrate cycle, alanine, aspartate and glutamate metabolism, pyrimidine metabolism, butanoate metabolism, pyruvate metabolism, fatty acid biosynthesis, valine, leucine and isoleucine degradation.

**Conclusion:** The use of metabolomics research methods to detect urine organic acids of children with GDD/ID can discover differential metabolites, which might be valuable for future research on the etiology, pathogenesis, prognosis and possible interventions of GDD/ID. The significantly altered differential metabolites indicators could therefore be potential diagnostic biomarkers for GDD/ID.

## 1 Introduction

Intellectual disability (ID) is a group of disorders characterized by cognitive impairment (IQ < 70), and social adjustment deficits that begin before the age of 18 years (Van Bokhoven, 2011). The term global developmental delay (GDD) is applicable for the children aged<5 years (Shevell, 2010).The prevalence of the ID in the population is about 1–3%, any factor that affects the brain development of children can lead to different degrees of GDD/ID in children (Maulik et al., 2011; Mullen et al., 2013). GDD/ID can seriously affect the quality of life of children and bring heavy economic burden to the family and society. Therefore, early etiology diagnosis and early intervention are of great significance for improving the prognosis of children with GDD/ID (Moeschler and Shevell, 2014).

The etiology of intellectual disabilities is very complex, which can be divided into non-genetic etiology and genetic etiology (O’Byrne et al., 2016). The genetic etiology accounts for about 2/3, including chromosomal abnormalities, monogenic diseases, polygenic diseases/epigenetic abnormalities, inherited metabolic diseases, etc (Shao et al., 2008; Carvill and Mefford, 2015; Chiurazzi and Pirozzi, 2016; Wang et al., 2016). The prevalence of these individuals carrying susceptible or disease-causing genes and loci is often higher than that of people with normal genetic material, but genetic risk factors alone are not sufficient to explain the complex pathogenesis and constitute a single cause of GDD/ID (Moeschler and Shevell, 2014).

At present, the genetic metabolic diseases that can be diagnosed account for 1–5% of the etiology of intellectual disabilities (García-Cazorla et al., 2009; van Karnebeek, 2014). With the development of diagnostic technology and gene analysis technology, the number of diagnosable and treatable genetic and metabolic diseases is increasing, which brings hope to the treatment of the etiology of children with GDD/ID. However, for developing countries, especially at the grassroots level or in rural areas, genetic testing is very expensive, and not everyone with GDD/ID can afford it. On the contrary, the cost of genetic metabolic tests is relatively low, and it is suitable for more patients as a basic test to find the etiology of GDD/ID patients. Genetic metabolic tests can help 1–5% of patients with GDD/ID identify the cause and potentially lead to effective treatment. For the remaining more than 95% of patients with GDD/ID, we can find differential metabolites to help patients find possible causes and provide possibilities for the next treatment.

Generally speaking, similar to children with Autism spectrum disorders (ASD), microbial metabolites, niacin metabolism, mitochondrial-related metabolites and amino acid metabolites are the most common problems in children with GDD/ID (Chen et al., 2019). These problems indicate the complexity of metabolic disorders and etiology in GDD/ID patients, leading to the exploration of building models for multivariate analysis. Related researches with large sample size on children have rarely been reported. The aims of this study were to identify metabolic signatures of GDD/ID as well as to find differential metabolites for possible etiologies and pathogenesis of GDD/ID. Thus, we undertook a retrospective review of all urine organic acid screening for the cases with GDD/ID referred to our department of pediatric neurology to determine the value of urine organic acid changes in the GDD/ID diseases, and to explore possible etiologies and pathogenesis of GDD/ID.

## 2 Materials and Methods

### 2.1 Participants

This retrospective study involved 1,253 cases who had GDD/ID and 100 typically developing children (TD) over the period from October 2015 to January 2021. Cases in the GDD/ID group were enrolled from the Xiangya Hospital, Central South University. Firstly, we screened positive results that could identify the cause of GDD/ID from 1,253 cases diagnosed with GDD/ID. Secondly, we screened the major differential metabolites and evaluated the diagnostic levels of differential metabolites in 132 urine organic acids from the remaining 1,230 negative results (863 GDD cases, 367 ID cases) that could not identify the cause of GDD/ID and 100 typically developing children (TD) (Supplementary Excel S1).

This study was approved by the Institutional Ethics Committee of Xiangya Hospital, Central South University. Both informed and written consents were obtained from the parents and/or legal guardians for study participation. Inclusion criteria for GDD/ID were as defined by the Diagnostic and Statistical Manual of Mental Disorders, Fifth Edition (DSM-V).

Exclusion criteria were: 1) presence of other diseases that may affect the development of the nervous system (e.g., intrauterine distress, history of hypoxia at birth, pathological jaundice, intracranial bleeding, perinatal infection, central nervous system infection, poisoning, and trauma history); 2) presence of chromosomal abnormalities, monogenic diseases, polygenic diseases which can cause GDD/ID. All participants were examined by senior pediatric neurologists. TD cases were enrolled from Children’s Health Clinic, Xiangya Hospital, Central South University.

### 2.2 Urine Samples Collection and Testing Method

Several precautions were strictly followed both before and after sampling to ensure specimen quality. The precautions and sampling steps were: 1) We took 10–20 ml of midstream urine in the morning, immersed 3 pieces of filter paper (the size was 5*5 cm) into the urine completely, took them out and dried them completely and put them in a specimen bag for inspection. Thereafter, we refrigerated it. Heating and drying were not allowed. 2) The qualified 3 specimens for each case were uniformly soaked and dried in urine filter paper sheets. 3) Samples were protected from the light and moisture and stored in refrigerator at 2–8°C for ≤ 14 days. 4) We rejected samples whose diaper infiltration area was too small, the specimen was contaminated or damaged, or moldy. The samples were collected from the inpatient or outpatient setting to ensure that external factors do not affect the samples. They were tested at the KingMed Diagnostics Laboratory (http://www.kingmed.com.cn/) (Guangzhou KingMed Diagnostics Group Co., Ltd, China). Gas chromatography/mass spectrometry (GC/MS) technology was performed for detection of organic acids. (See Supplementary Word S1 for specific test methods).

### 2.3 Clinical Data Collection and Follow up

Information concerning study population characteristics was obtained from the Xiangya Hospital, Central South University electronic medical record system. Follow-up information was collected through regular clinic, WeChat and telephone communication. All assessments of children’s condition, daily life, and eating habits were provided by specialized clinicians and parents.

### 2.4 Data Processing, Modeling and Analysis

All urine organic acid test results were collected and entered into a spreadsheet database (Excel for Windows 10, Microsoft, China). Each item included identification number, name, sex, date of birth, test time, diagnosis, test report and results. R language script and IBM SPSS Statistics 22.0 software (IBM, Armonk, NY, United States) were used to analyse the data. Variables conforming to the normal distribution were expressed as mean ± standard deviation, and measurement data not conforming to the normal distribution was expressed as median or quaternary. Two-sample independent *t*-test was used for comparison. Categorical data were summarized in the form of frequencies and proportions, and analyzed with the Chi-square test or Fisher’s exact test where applicable. The level of significance was set at *p* ≤ 0.05.

Non-supervisory principal component analysis (PCA) and orthogonal partial least squares discriminant analysis (OLPS-DA) were used to compare the differences of major organic acids in urine between the affected children and TD group. We used a criteria of VIP>1.0 and *p* < 0.05 to filter out candidate metabolites. The differences of some organic acids in urine of each group were analyzed by heatmap. Metabo Analyst5.0, KEGG database, Metlin database and HMDB database were used to analyze relevant data and metabolic pathways. Receivers operating characteristic curve (ROC) and Youden index were used to determine the optimal diagnostic cut-off point of urine organic acids.

## 3 Results

### 3.1 Patients With Global Developmental Delay/Intellectual Disability Whose Causes Were Determined by Urine Organic Acid Detection

A total of 1330 participants were enrolled including 863 GDD cases (GDD group), 367 ID cases (ID group) and 100 TD children (TD group). A total of 1253 GDD/ID patients were enrolled including 23 patients with GDD/ID whose causes were determined by urine organic acid detection and 1230 patients with GDD/ID whose causes were unknown. The cause of 1.8% patients with GDD/ID could be identified by urine organic acid tests, including 7 cases of methylmalonate, 4 cases of glycerol kinase deficiency, 3 cases of glutaric aciduria, 2 cases of tyrosine metabolism disorder, 2 cases of Maple syrup urine disease. 2 cases of propionate aciduria, 1 case of ethyl malonate aciduria, 1 case of phenylketonuria, and 1 case of hyperlactic aciduria.

### 3.2 Results of the Principal Component Analysis and Orthogonal Partial Least Squares Discriminant Analysis of Urine Organic Acids

**FIGURE 1:**
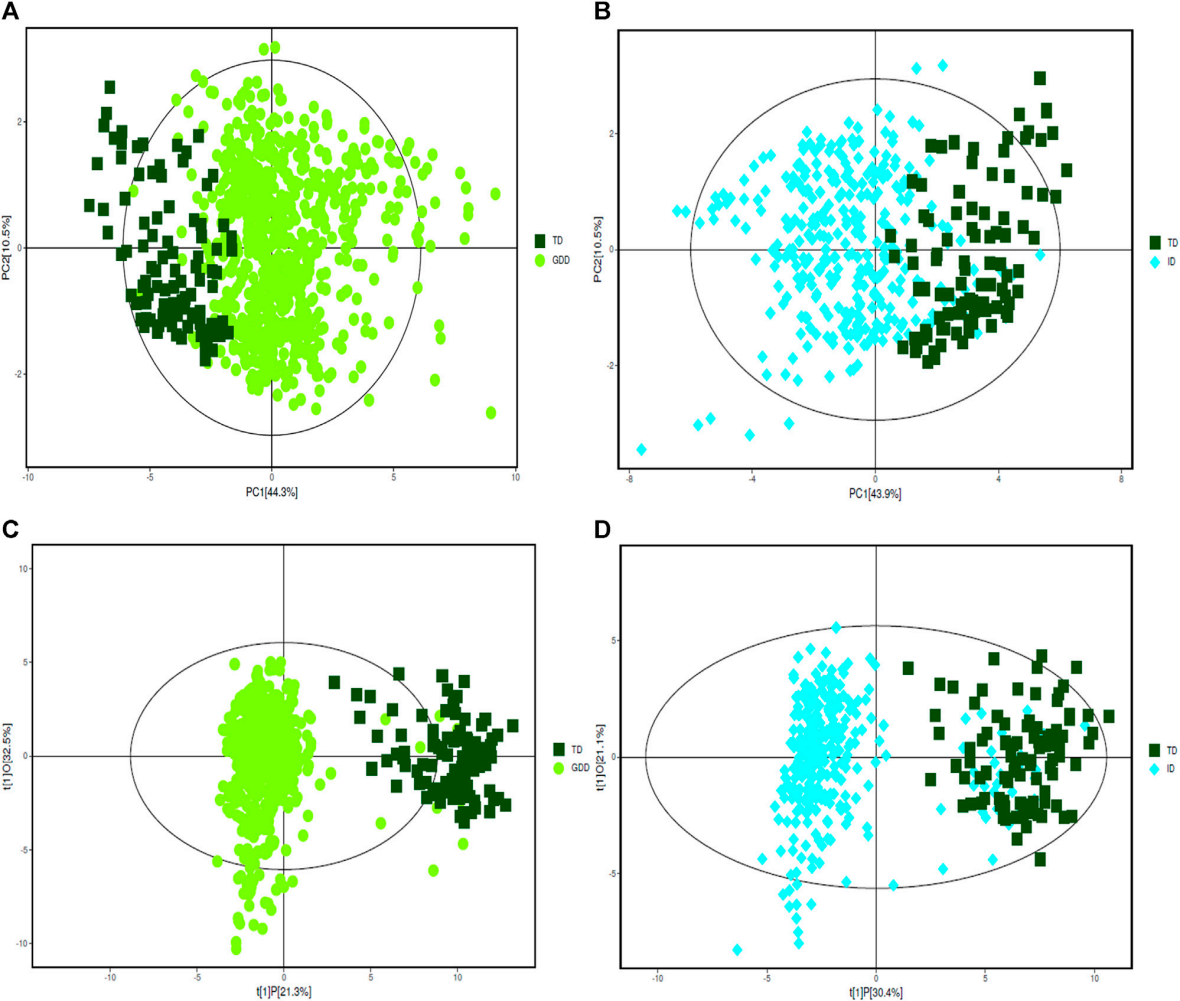
PCA and OPLS-DA score plots. **(A)** The PCA score plot of GDD group. **(B)** The PCA score plot of ID group. **(C)** The OPLS-DA score plot of GDD group. **(D)** The OPLS-DA score plot of ID group.

The urinary organic acids values of the cases in the GDD and ID groups were compared with those in the TD group, and the difference was statistically significant (*p* < 0.05). One hundred and thirty two kinds of organic acids were compared and the difference was statistically significant for 57 organic acids (Supplementary Table S1). Except for palmitic acid, the other 56 urinary organic acid values in GDD and ID groups were all higher than that of the TD group. The PCA results of the three groups with all acids are shown on Figures 1A,B. From the figure, we see that there are differences between the GDD group and the ID group and the TD group respectively. Most of the sample points fall within the 95% confidence interval of Hotelling’s T2 ellipse. (Supplementary Table S2). In order to eliminate the noise information that is not related to the classification, and also to obtain the related metabolite information that causes significant differences between the two groups, we used orthogonal partial least square discriminant analysis (OPLS-DA) to filter the signals that are not related to the model classification. The OPLS-DA results of the three groups are summarized in Supplementary Figures 1C,D and Supplementary Table S3. Both the GDD and the ID models had high explanatory and predictive rates, indicating that this model can distinguish between the two groups. OPLS-DA permutation plot results of GDD and ID groups showed no overfitting, and subsequent analysis of differential metabolite results was reliable. (Supplementary Figure S1)

### 3.3 Potential Marker Metabolites

According to the coefficient confidence interval of each variable and the VIP value of the variable weight, the meaningful difference variable (*p* < 0.05, VIP>1.0) was selected, that is, the specific biomarker. The identified potential marker metabolites are listed in Table 1. The comparisons of their levels between both ID and GDD groups versus TD group are also shown in Table 1. Compared with the TD group, the differential metabolites of the GDD group and the ID group had the same trend. Twenty four different metabolites were screened out in the GDD group, and 25 different metabolites were screened out in the ID group (VIP>1.0, *p* < 0.01). (Supplementary Excel S2) Except for palmitic acid, metabolites in GDD group and ID group were up-regulated, compared with TD group.

**TABLE 1:** Potential marker-metabolites found in urine organic acid screening.

	GDD				ID			
NO.	Metabolite	Differentiation for GDD samples	*p*-value	VIP	Metabolite	Differentiation for ID samples	*p*-value	VIP
1	Methyl citric acid (1)	↑	1.65E-93	1.76	Methyl citric acid (2)	↑	3.51E-63	1.59
2	Methyl citric acid (2)	↑	1.43E-93	1.76	Methyl citric acid (1)	↑	5.01E-54	1.59
3	Malic acid - 3	↑	2.32E-77	1.51	Malic acid	↑	1.38E-53	1.48
4	2-Ketoglutaric acid	↑	7.83E-31	1.49	Orotic acid	↑	2.27E-50	1.48
5	2-ketone - isocaproic acid	↑	5.88E-89	1.48	Glycolic acid	↑	2.35E-36	1.48
6	2-deoxy-4-hydroxyacetoacetic acid	↑	4.26E-60	1.47	2-deoxy-4-hydroxyacetoacetic acid	↑	3.26E-31	1.44
7	Orotic acid	↑	7.89E-87	1.43	Octenedioic acid	↑	9.46E-52	1.43
8	2-hydroxyadipate	↑	1.07E-78	1.43	Palmitic acid	↓	3.11E-42	1.43
9	3-hydroxy octanoic acid	↑	2.38E-75	1.40	Uracil	↑	1.53E-40	1.42
10	Uracil	↑	1.12E-70	1.40	N-acetyl-aspartic acid	↑	1.72E-44	1.41
11	3-hydroxyisobutyric acid	↑	1.23E-49	1.40	2- hydroxyl phthalate	↑	7.11E-50	1.41
12	2-hydroxyadipate	↑	1.57E-74	1.38	2-Ketoglutaric acid	↑	2.06E-51	1.36
13	Octenedioic acid	↑	8.78E-72	1.34	2-hydroxyadipate	↑	1.24E-43	1.36
14	N-acetyl-aspartic acid	↑	5.57E-74	1.33	3-hydroxy octanoic acid	↑	2.14E-48	1.34
15	Glycolic acid	↑	9.77E-54	1.30	2-ketone - isocaproic acid	↑	5.73E-17	1.34
16	3-hydroxypropionic acid	↑	3.06E-59	1.29	3-hydroxypropionic acid	↑	6.79E-29	1.32
17	Isocotonic acid	↑	5.3E-84	1.29	3-hydroxyisobutyric acid	↑	4.39E-25	1.28
18	Palmitic acid	↓	1.89E-41	1.24	Pimelic acid	↑	8.86E-34	1.26
19	Pimelic acid	↑	3.24E-55	1.23	3- hydroxy quinodioic acid	↑	2.69E-37	1.24
20	3-hydroxyglutaric acid	↑	4.36E-70	1.21	3, 6-epoxy-dodecanedioid	↑	8.96E-38	1.22
21	3, 6-epoxy-dodecanedioid	↑	2.21E-51	1.17	Isocotonic acid	↑	1.42E-28	1.17
22	3- hydroxy quinodioic acid	↑	2.18E-56	1.15	Mesaconic acid	↑	3.93E-41	1.14
23	Phosphate	↑	2.75E-44	1.02	3-hydroxyglutaric acid	↑	7.99E-27	1.12
24	Uric acid	↑	1.48E-05	1.02	2-hydroxyglutaric acid	↑	3.74E-37	1.06
25					5-hydroxy-methyl-2-furfuric acid	↑	3.32E-09	1.02

Methyl citric acid (1) and Methyl citric acid (2) are the same substance with different fronting time in the detection process.

### 3.4 Heatmap Analysis of Differential Metabolites

**FIGURE 2:**
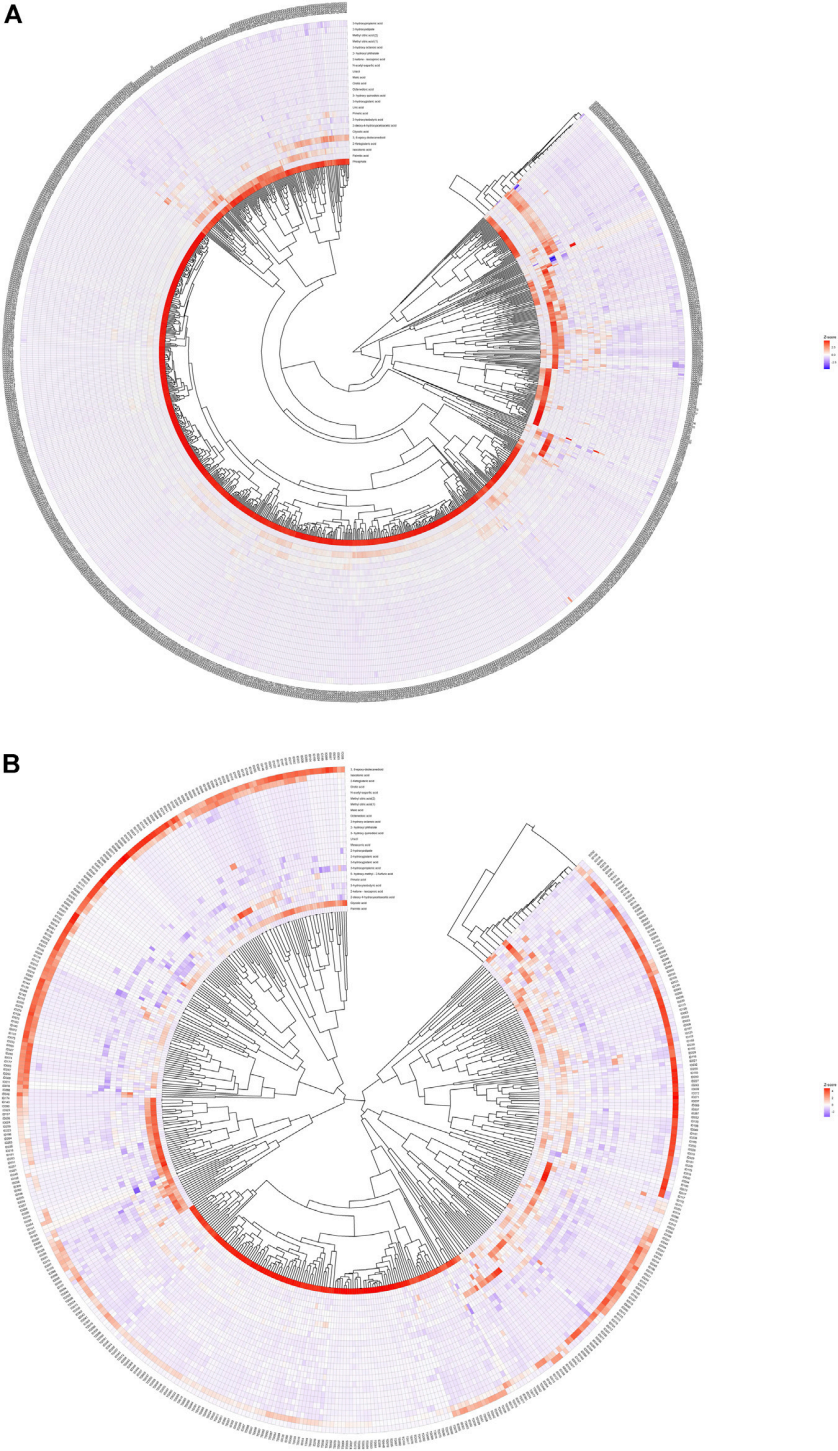
Heatmap analysis of differential metabolites. **(A)** is heatmap analysis of differential metabolites in GDD group. **(B)** is heatmap analysis of differential metabolites in ID group.

We calculated the Euclidean Distance Matrix for the quantitative values of the differential metabolites, clustered the differential metabolites by the complete linkage method, and displayed them in the form of heatmap (Figure 2). The outer circle of the circular heatmap represent different samples from GDD and TD groups, ID and TD groups, while the sides of the circular heatmap represent different metabolites. The color blocks at different positions represent the relative expression of metabolites at the corresponding positions. The redder the color, the higher the relative expression content, and the bluer the color, the lower the relative expression content. The top five identified possible biomarkers were palmitic acid, isocotonic acid, ethyl hydroxypropionic acid, ethylmalonic acid, and glycolic acid.

### 3.5 Correlation Analysis of Different Metabolites

**FIGURE 3:**
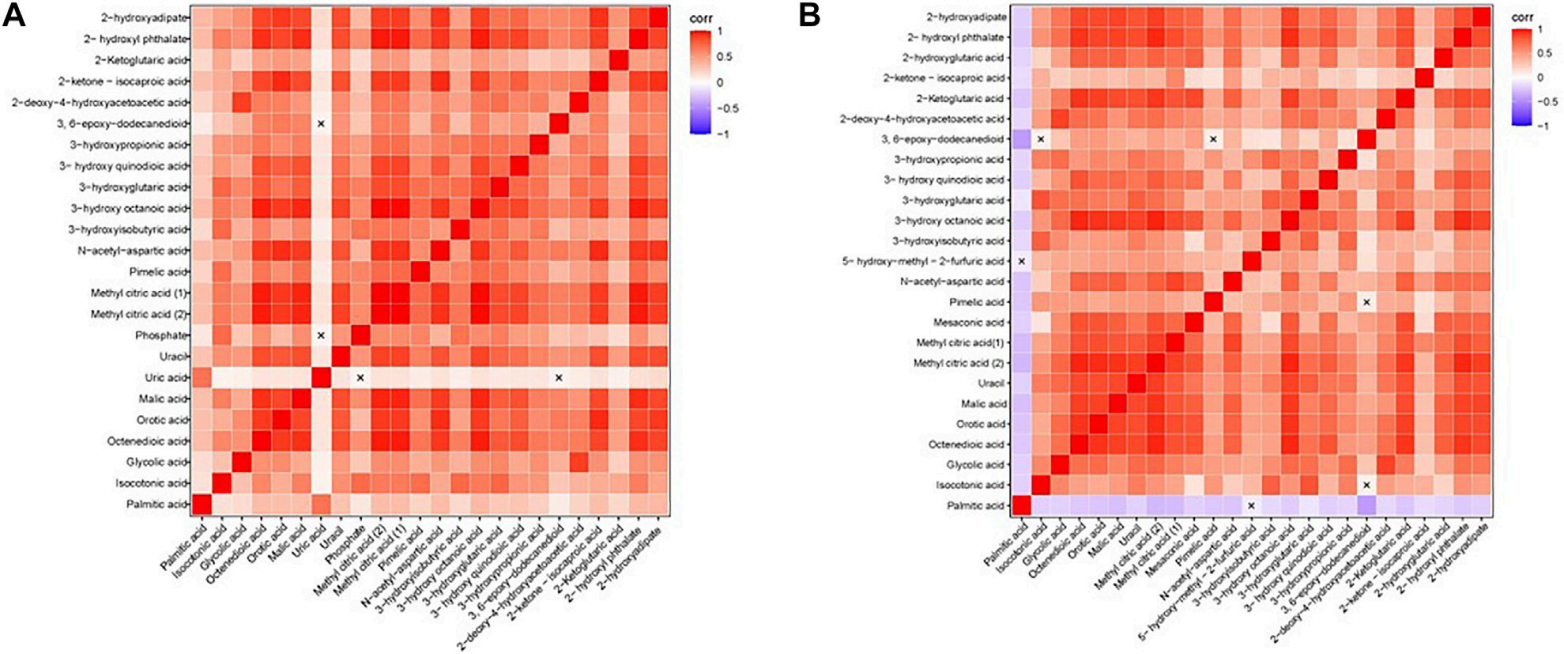
Heatmap of correlation analysis. **(A)** The Heatmap of correlation analysis of GDD group. **(B)** The Heatmap of correlation analysis of ID group. The horizontal and vertical coordinates in the figures represent comparison of different metabolites. The color blocks at different positions represent the correlation coefficients between the metabolites at the corresponding positions. Red indicates positive correlation, blue indicates negative correlation, and non-significant correlations are marked with a cross (×).

We calculated the correlation coefficient of the quantitative value of differential metabolites by Pearson method and presented it in the form of heatmap (Figure 3). We conducted correlation analysis on multiple differential metabolites to measure the degree of correlation between two differential metabolites, and the degree of correlation between two variables was represented by the correlation coefficient r. In the case of positive correlation, the value of r was between 0 and 1; in the case of negative correlation, the value of r was between −1 and 0. The closer the absolute value of r was to 1, the stronger the correlation between the two differential metabolites; the closer the absolute value of r was to 0, the weaker the correlation between the quantity variable.

### 3.6 Cut-Off Values of Differential Metabolites Between Global Developmental Delay Group and Intellectual Disability Group

The cut-off values of the different metabolites in the GDD and ID groups were calculated and sorted by using ROC curve and Youden index. Among the different metabolites in the GDD group, palmitic acid’s cut-off value was 7.190, area under the cut-off value curve (AUC) was 0.985, glycolic acid’s cut-off value was 2.210, AUC was 0.953, and 3-hydroxyisobutyric acid’s cut-off value was 2.315, AUC was 0.948. Among the different metabolites in the ID group, palmitic acid’s cut-off value was 5.690, AUC was 0.963, glycolic acid’s cut-off value was 2.810, AUC was 0.954, and 3-hydroxyisobutyric acid’s cut-off value was 2.330, AUC was 0.916. It can be seen from the Table 2 that the cut-off values of palmitic acid, glycolic acid, and 3-hydroxyisobutyric acid had high value for early identification of GDD/ID. The specificities of these three substances in the GDD group were 98.0, 90.0, and 91.0%, respectively, and for ID group were 99.0, 96.0, and 91.0%, respectively (Table 2). Supplementary Figure S2 shows the ROC curve of the cut-off value of palmitic acid, glycolic acid, 3-hydroxyisobutyric acid in the GDD group and the ID group. It can be seen from the figure that the AUC of all differential metabolites was higher than 0.9.

**TABLE 2:** Cut-off values of different metabolites in GDD group and ID group.

Differential metabolites	Group	Cut-off value	AUC	Sensitivity	Specificity	Youden index
Palmitic acid	GDD group	7.190	0.985	96.1	98.0	0.941
	ID group	5.690	0.963	88.0	99.0	0.870
Glycolic acid	GDD group	2.210	0.953	88.6	90.0	0.786
	ID group	2.810	0.954	84.7	96.0	0.807
3-hydroxyisobutyric acid	GDD group	2.315	0.948	90.3	91.0	0.813
	ID group	2.330	0.916	79.6	91.0	0.706

Abbreviations: AUC, area under the cut-off value curve; GDD, global developmental delay; ID, intellectual disability.

### 3.7 Pathway Analysis of Differential Metabolites

**FIGURE 4:**
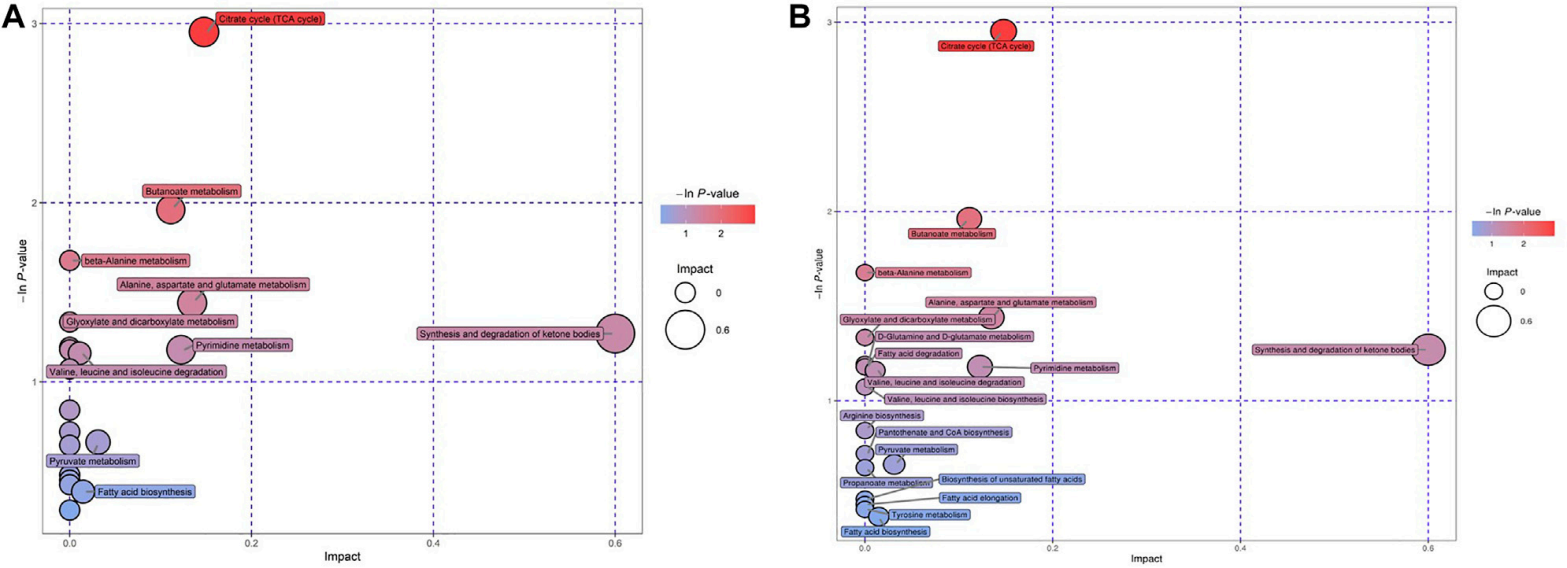
**(A)** Bubble charts of the Pathway analysis of differential metabolites in GDD group. **(B)** Bubble charts of the Pathway analysis of differential metabolites in ID group.

We tried to use bubble charts to show the possible related metabolic pathways (Figure 4). Each bubble in the bubble diagram represented a metabolic pathway. The abscissa of the bubble and the size of the bubble represented the size of the influence factor of the pathway in the topological analysis. The larger the size, the greater the influence factor. The vertical coordinate where the bubbles were located and the color of the bubbles represented the *p* value of enrichment analysis (take the negative natural logarithm, i.e. –In (P)). The darker the color, the smaller the *p* value and the more significant the enrichment degree. After bubble chart analysis, the pathway analysis of the differential metabolites in the GDD and ID groups were basically the same. According to the order of impact value and–In (P) value, the top 8 pathways were the synthesis and degradation of ketone bodies, citrate cycle (TCA cycle), alanine, aspartate and glutamate metabolism, pyrimidine metabolism, butanoate metabolism, pyruvate metabolism, fatty acid biosynthesis, valine, leucine and isoleucine degradation. This diverse distribution suggest that these organic acids may act on a variety of metabolic pathways and reflects the complexity of metabolic abnormalities in GDD/ID.

## 4 Discussion

Urine organic acid detection can identify the etiology of some patients with GDD/ID. In our study, 1.8% of patients with GDD/ID could be identified by urine organic acid detection. This is consistent with previous reports (García-Cazorla et al., 2009; van Karnebeek, 2014). In order to identify the metabolic signatures of GDD/ID and look for organic acids in urine that could be valuable for future research on the etiology and pathogenesis of GDD/ID, and prognosticate diseases clinically, PCA and OPLS-DA algorithms were used to analyze urine organic acids data. The modeling was performed on the basis of all 132 urine organic acids, among which 24 different metabolites in GDD group and 25 differential metabolites in ID group were identified. To go a step further, we selected top 3 urine organic acids as differential metabolites with the most diagnostic value. The amount of glycolic acid and 3-hydroxyisobutyric acid were significantly higher in both GDD and ID groups as compared to TD group while the amount of palmitic acid was significantly lower in both GDD and ID groups in comparison to TD group. These urine organic acids are involved in a variety of metabolic pathways including the synthesis and degradation of ketone bodies, citrate cycle (TCA cycle), alanine, aspartate and glutamate metabolism, pyrimidine metabolism, butanoate metabolism, pyruvate metabolism, fatty acid biosynthesis, valine, leucine and isoleucine degradation. We selected differential metabolites with the most diagnostic value in discussion part since they made contributions in diagnosis and disease treatment.

### 4.1 Complex Relationships Among Urinary Organic Acids and Global Developmental Delay/Intellectual Disability Pathogenesis

People constantly receive and respond to external or internal stimuli, and these experiences are learned and memorized in their brains. For this learning and memory process, nerve cells in the brain undergo enormous molecular and cellular changes, not only in the input -output -related local subcellular compartments but also in the central nucleus (Yu et al., 2011). The metabolic disorder of urine organic acid is caused by the lack of certain enzyme, which leads to the accumulation of related carboxylic acid and its metabolites, resulting in metabolic acidosis and damage of brain, liver, kidney, heart, bone marrow and other organs (Gabbi et al., 2017). Due to the accumulation of toxic metabolites, energy synthesis disorders, mitochondrial dysfunction, neuronal cell apoptosis, cytoskeletal phosphorylation changes and myelination disorders, patients' brain nerve structure damage, ganglioside and synaptic plasticity abnormalities, resulting in decreased learning and memory ability, and even intellectual disabilities (Gabbi et al., 2017).

The heatmap generated from our analysis of urinary organic acids showed the complex relationship among these compounds in GDD/ID. Several organic acids were in the same pathway, whereas others were involved in multiple pathways. To date, the metabolites that have been explored as possible GDD/ID biomarkers include: methylmalonic acid, propionic acid, methyl citrate, propionyl carnitine, propionyl glycine (Horster and Hoffmann, 2004; Wongkittichote et al., 2017), glutaric acid, 3-hydroxyglutaric acid (van Karnebeek and Stockler, 2012; Hoytema van Konijnenburg et al., 2021), 4-hydroxybutyric acid, γ-aminobutyric acid (Li et al., 2015), 3-hydroxyisobutyric acid (van Karnebeek and Stockler, 2012), 2-hydroxyglutaric acid (Kranendijk et al., 2012). The exact pathogenesis of the accumulation of these metabolites leading to GDD/ID is still unclear. The currently known pathogenesis mainly includes: energy metabolism disorders that affect structure and function of the brain (Flippo and Strack, 2017), energy substance supply disorders, neurotransmitter abnormalities that affect neurotransmission (Lim et al., 2020), the accumulation of harmful metabolic substrates or the inability of normal utilization of metabolites affecting the formation of myelin or lead to dysfunctional myelin (Marques and Saftig, 2019).

### 4.2 Notable Changes in Urinary Organic Acid Levels in Global Developmental Delay/Intellectual Disability Patients

The OPLS-DA score plot showed a clear difference among the distribution characteristics of metabolites between GDD/ID cases and TD children. Our analyses showed that 5 urine organic acids had significant differences between GDD/ID cases and TD children and thus, could be valuable for future research on the etiology and pathogenesis of GDD/ID, and prognosticate diseases clinically.

The GDD group had higher levels of methyl citric acid, malic acid, 2-ketoglutaric acid, 2-ketone-isocaproic acid and 2-deoxy-4-hydroxyacetoacetic acid compared to TD children. Besides, the ID group had higher levels of methyl citric acid, malic acid, orotic acid, glycolic acid and 2-deoxy-4-hydroxyacetoacetic acid compared to TD children. On the contrary, both GDD group and ID group had decreased amounts of palmitic acid. These metabolites are associated with multiple biochemical processes (Koulman et al., 2009). The increase in orotic acid is more common in urea circulation disorders (UCDs), which leads to the dysfunction of the central nervous system (Savy et al., 2018). In our study, children with GDD/ID had a higher level of orotic acid in their urine, which was speculated to be related to UCDs, hyperammonemia, amino acid metabolism disorders, and ultimately cause damage to the nervous system. Palmitic acid is an intermediate product of fat acid synthesis and metabolism. Many key proteins related to neural development can be modified and processed by palmitoylation, which play important regulatory roles in the growth, polarization, and establishment of neural networks of neurons (Globa and Bamji, 2017). We speculate that reduction in palmitic acid content affects the palmitoylation processing level, thereby causing damage to the development of the nervous system.

## 5 Conclusion

In this study, we screened out 23 positive cases with clear etiology of GDD/ID from 1253 urine reports of organic acids diagnosed with GDD/ID, accounting for 1.8% of the total. PCA and OLPS-DA analysis methods were used to develop models to distinguish the remaining 1230 negative results with unclear etiology of GDD/ID from TD children and to detect major differential metabolites. By a voting mechanism, 24 different metabolites were screened out in the GDD group, and 25 different metabolites were screened out in the ID group, which were successfully identified as causes of GDD/ID. These differential metabolites were distributed across a wide variety of metabolic pathways, indicating the complicated mechanism behind GDD/ID.

For GDD/ID patients whose cause cannot be found, we can search for the possible cause of the disease and judge its prognosis through the discovered differential metabolites. If GDD/ID can be identified early by these discovered differential metabolites, these patients can be treated earlier by supplementing the lack of metabolites, expelling the excess metabolites in the body, and forbidding ingesting contraindicated foods or drugs.

In summary, the use of metabolomics research methods to detect urine organic acids of children with GDD/ID can discover differential metabolites, which might be valuable for future research on the etiology, pathogenesis, prognosis and possible interventions of GDD/ID.

## 6 Limitation

We used the VIP scores to predict the differential metabolites of 132 urinary organic acids in 1230 children with GDD/ID. This method has resulted in the discovery of a group of biochemically different substances in 132 urine organic acids, which may help to distinguish individuals at risk for GDD/ID. Since our data originated from a single-center, it is difficult to determine how generalizable these predictive features will be in a broader population. More centers and larger studies are needed to evaluate and refine a signature for clinical diagnosis. So far, several of the metabolites identified in these features point to biological mechanisms that have been previously thought to play a role in the etiology of GDD/ID. Our differential metabolites might represent a portion of metabolic changes that are crucial metabolic endpoint for the diagnosis of GDD/ID.

## Data Availability

The original contributions presented in the study are included in the article/Supplementary Material, further inquiries can be directed to the corresponding author.
