# Supplementary material for: Urine Organic Acids as Metabolic Indicators for Global Developmental Delay/Intellectual Disability in Chinese Children
Source: Front Mol Biosci. 2021 Dec 22;8:792319. doi: 10.3389/fmolb.2021.792319 (PMC8757376; doi:10.3389/fmolb.2021.792319)
Supplement: Supplementary file 2 [file Table5.DOCX]

**GC/MS test methods**

1. Instrument model: Shimadzu GC-MS QP2020

2. The precautions and sampling steps were:

(1) We took 10-20 ml of midstream urine in the morning, immersed 3 pieces of filter papers (the size was 5*5 cm) into the urine completely, took them out and dried them completely and put them in a specimen bag for inspection. Thereafter, we refrigerated them. Heating and drying were not allowed.

(2) The qualified 3 specimens for each case were uniformly soaked and dried in urine filter paper sheets.

(3) Samples were protected from the light and moisture, and stored in refrigerator at 2-8℃ for < 14 days.

(4) We rejected samples whose diaper infiltration area was too small, the specimen was contaminated or damaged, or moldy.

3. Sample processing steps before urine organic acid detection:

(1) Urease was taken into a lidless centrifugal tube and loaded into a syringe.

(2) A piece of dry urine paper was put into the syringe, ultra-pure water with a glass adjustable liquidizer was added to make the filter paper very wet. The filter paper was squeezed to make it dry with the plunger of the syringe, and the complex solution was oscillated and mixed evenly.

(3) The complex solution was transferred into a Hitachi cup, the urinary creatinine content was measured, and the lipless centrifugal tube was put into a drying box for the reaction.

(4) The solution of the reacted liquid was transferred to another covered centrifugal tube. The internal standard solution, ultra-pure water, hydroxylamine hydrochloride and NaOH solution were mixed together well and allowed to react at room temperature.

(5) Pure hydrochloric acid was then added, mixed well, then allowed to stand still. The pH test paper was used to test the pH value of the sample to ensure that the sample was acidic.

(6) Ethyl acetate was added, shaken thoroughly, and then centrifuged. The supernatant was transferred to a clean centrifugal tube, and the operation was repeated once.

7) The extracted liquid was placed into a nitrogen blow-dryer for drying it.

8) The derivatization reagent was added, and then placed in drying oven for the chemical reaction to occur. The reaction solution was then transferred into the sample bottle, and thereafter tested on the machine.

**Derivatization reagents and GC/MS parameters**

Standard substance: The hydrocarbon mixture (C10–C26, even numbers).

Internal standard solution: Heptadecanoic acid, tetracosanoic acid and tropic acid.

Derivatization reagent: N-, O-bis (trimethylsilyl)-trifluoroacetamide(BSTFA) and trimethylchlorosilane (TMCS).

**Instrument and equipment parameters**

**Chromatographic column**: The capillary column was a fused-silica DB-5 one (50 m×0.25 mm i.d.) with a 0.25-μm film thickness of 5% phenylmethylsilicone.

**Mass spectrum parameters**: Mass spectra were obtained by standard electron impact ionization scanning from m/z 50 to m/z 600 at a rate of 0.4 s/cycle.

**Programmed temperature regulation**: The temperature program was started at 100℃ with an initial holding for 2 min, and was then increased at a rate of 8℃/min to 280℃ with a final holding for 10min. The temperatures of the injection port and transfer line were both 270℃. Flow rate of the helium carrier was 1.4 ml/min, and the linear velocity was 40.0 m/s.

**Injection volume**: 1.0μl of the final derivatized aliquot was injected into the GC/MS and analyzed in the split mode (10:1).
