# Supplementary material for: Urine Organic Acids as Metabolic Indicators for Global Developmental Delay/Intellectual Disability in Chinese Children
Source: Front Mol Biosci. 2021 Dec 22;8:792319. doi: 10.3389/fmolb.2021.792319 (PMC8757376; doi:10.3389/fmolb.2021.792319)
Supplement: Supplementary file 3 [file Table3.DOCX]

**A**

**B**


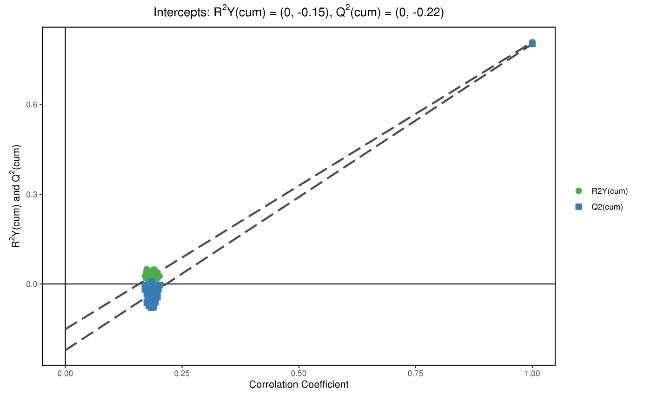

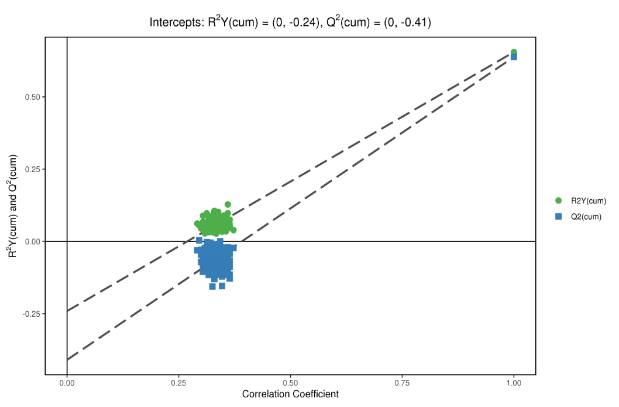


**Supplementary Figure 1** **OPLS-DA permutation plots. (A)** The OPLS-DA permutation plot of GDD group. **(B)** The OPLS-DA permutation plot of ID group.


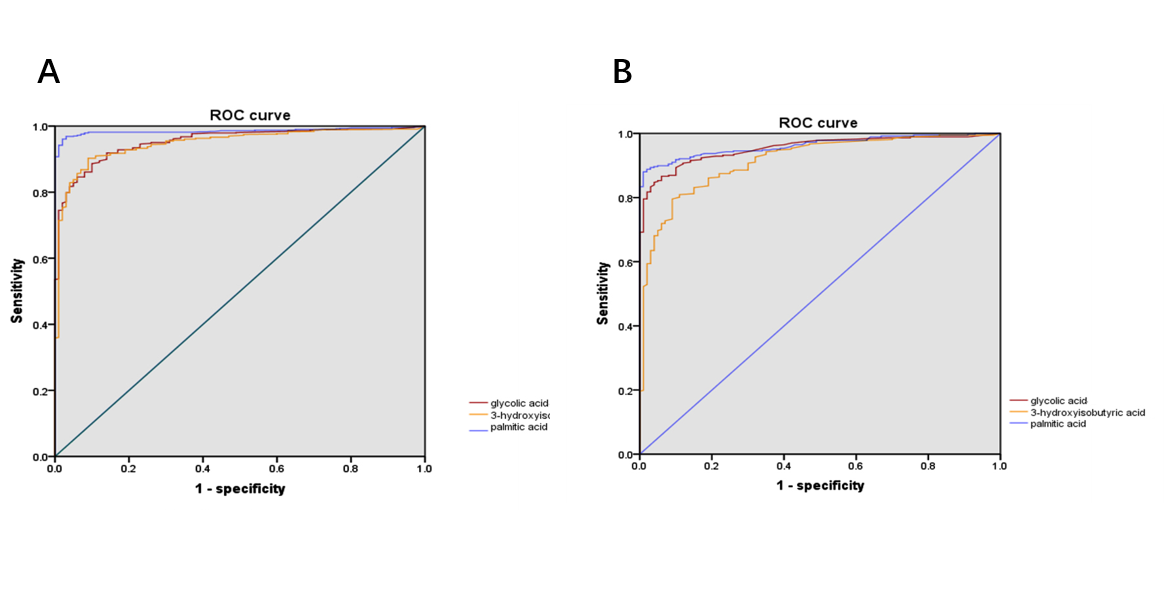


**Supplementary Figure 2 (A) ROC curve of the cut-off value of different metabolites in GDD group. (**B) **ROC curve of the cut-off value of different metabolites in ID group.**
