# Supplementary material for: Urine Organic Acids as Metabolic Indicators for Global Developmental Delay/Intellectual Disability in Chinese Children
Source: Front Mol Biosci. 2021 Dec 22;8:792319. doi: 10.3389/fmolb.2021.792319 (PMC8757376; doi:10.3389/fmolb.2021.792319)
Supplement: Supplementary file 5 [file Table4.DOCX]

**Supplementary Table 1**

**Results of the comparison of urine organic acids values**

|  | **TD group** | **GDD group** | | | **ID group** | | |
| --- | --- | --- | --- | --- | --- | --- | --- |
| **Compound name** | **Mean** | **Mean** | **VIP** | **P-Value** | **Mean** | **VIP** | **P-Value** |
| Palmitic acid | 21.84 | 6.11 | 1.24 | 1.89E-41 | 5.58 | 1.43 | 3.11E-42 |
| Isocotonic acid | 4.28 | 21.58 | 1.29 | 5.30E-84 | 14.40 | 1.17 | 1.42E-28 |
| Ethyl hydroxypropionic acid | 0.97 | 3.19 | 0.47 | 1.94E-33 | 2.19 | 0.44 | 4.84E-16 |
| Ethylmalonic acid | 0.71 | 3.35 | 0.65 | 3.58E-32 | 2.36 | 0.73 | 4.69E-26 |
| Glycolic acid | 1.17 | 11.04 | 1.30 | 9.77E-54 | 11.51 | 1.48 | 2.35E-36 |
| Octenedioic acid | 0.88 | 4.96 | 1.34 | 8.78E-72 | 3.52 | 1.43 | 9.46E-52 |
| Suberic acid | 0.96 | 4.66 | 0.67 | 1.25E-23 | 3.22 | 0.88 | 5.68E-17 |
| Vanillic acid | 1.30 | 4.88 | 0.63 | 0.00 | 2.63 | 0.77 | 0.00 |
| Glutaric acid | 0.84 | 4.45 | 0.60 | 3.85E-07 | 2.88 | 0.72 | 4.10E-20 |
| Lactic acid | 1.05 | 3.80 | 0.52 | 0.00 | 2.40 | 0.42 | 0.00 |
| Orotic acid | 0.47 | 4.74 | 1.43 | 7.89E-87 | 3.21 | 1.48 | 2.27E-50 |
| Azelaic acid | 2.21 | 5.41 | 0.30 | 1.48E-09 | 3.59 | 0.44 | 3.95E-06 |
| Malic acid | 0.49 | 4.82 | 1.51 | 2.32E-77 | 3.30 | 1.48 | 1.38E-53 |
| Urine vanilla mandelic acid | 16.11 | 35.07 | 0.45 | 1.13E-12 | 22.43 | 0.05 | 0.00 |
| Uric acid | 1.65 | 7.08 | 1.01 | 1.48E-05 | 3.87 | 0.99 | 2.64E-17 |
| Urea | 123.65 | 379.21 | 0.08 | 1.68E-16 | 289.56 | 0.00 | 0.00 |
| Uracil | 0.81 | 5.26 | 1.40 | 1.12E-70 | 3.61 | 1.42 | 1.53E-40 |
| Hippuric acid | 20.24 | 39.34 | 0.45 | 3.86E-09 | 41.94 | 0.64 | 3.41E-09 |
| Phosphoric acid | 23.63 | 146.13 | 1.02 | 2.75E-44 | 85.71 | 0.94 | 1.50E-17 |
| Aoi diacid | 1.03 | 8.36 | 0.66 | 0.00 | 4.57 | 0.68 | 0.00 |
| Methyl succinic acid | 1.02 | 3.25 | 0.34 | 6.43E-29 | 2.61 | 0.43 | 1.55E-11 |
| Methylphenidate-(2) | 0.48 | 5.29 | 1.76 | 1.43E-93 | 3.57 | 1.59 | 3.51E-63 |
| Methylphenidate(1) | 0.48 | 5.29 | 1.76 | 1.65E-93 | 3.70 | 1.59 | 5.01E-54 |
| Methylfumaic acid | 0.61 | 3.95 | 0.91 | 7.26E-57 | 2.84 | 1.14 | 3.93E-41 |
| Hexic acid | 0.96 | 4.43 | 0.62 | 9.58E-25 | 3.07 | 0.66 | 8.22E-14 |
| Amber acid | 6.90 | 10.75 | 0.06 | 0.00 | 5.02 | 0.66 | 0.01 |
| Arrhythmythic acid | 55.26 | 221.30 | 0.69 | 1.12E-52 | 147.41 | 0.64 | 1.60E-18 |
| Diacid | 1.45 | 7.34 | 1.23 | 3.24E-55 | 5.76 | 1.26 | 8.86E-34 |
| High vanilla acid | 5.90 | 15.11 | 0.83 | 3.28E-27 | 8.38 | 0.29 | 9.78E-05 |
| Foma acid | 1.10 | 4.18 | 0.64 | 9.41E-49 | 3.24 | 0.92 | 1.65E-34 |
| Oxalic acid | 1.60 | 6.34 | 0.85 | 1.51E-38 | 3.68 | 0.59 | 2.69E-13 |
| Acetone acid | 5.88 | 14.88 | 0.63 | 0.00 | 10.41 | 0.58 | 1.46E-06 |
| Benzoic acid | 0.95 | 2.43 | 0.64 | 2.31E-22 | 1.75 | 0.79 | 1.36E-12 |
| Sabbath acid | 1.53 | 5.78 | 0.49 | 1.51E-10 | 5.60 | 0.68 | 2.40E-07 |
| N-acetylmenosterine | 0.69 | 4.59 | 1.33 | 5.57E-74 | 3.35 | 1.41 | 1.72E-44 |
| 5-hydroxyl-methyl-2-arbiric acid | 1.70 | 6.14 | 0.98 | 6.53E-14 | 4.38 | 1.02 | 3.22E-09 |
| 4-hydroxybenzene acetic acid | 11.57 | 39.16 | 0.75 | 2.87E-32 | 23.65 | 0.49 | 7.64E-07 |
| 4-hydroxybenzene lactic acid | 1.95 | 10.72 | 0.81 | 0.00 | 4.58 | 0.86 | 2.08E-06 |
| 4-hydroxysalolic acid | 2.93 | 12.20 | 0.90 | 9.44E-29 | 8.68 | 0.87 | 1.82E-14 |
| 3-hydroxy-isoprenic acid | 1.20 | 4.43 | 0.75 | 9.87E-43 | 3.01 | 0.79 | 1.26E-29 |
| 3-hydroxyisbutyric acid | 1.21 | 8.80 | 1.40 | 1.23E-49 | 6.10 | 1.28 | 4.39E-25 |
| 3-hydroxysindic acid | 1.03 | 5.35 | 1.40 | 2.38E-75 | 3.69 | 1.34 | 2.14E-48 |
| 3-hydroxyquine | 1.49 | 7.19 | 1.21 | 4.36E-70 | 5.21 | 1.12 | 7.99E-27 |
| 3-hydroxyfloweric acid | 0.86 | 5.00 | 1.14 | 2.17E-56 | 3.32 | 1.23 | 2.69E-37 |
| 3-hydroxybutyric acid | 1.08 | 20.59 | 0.57 | 1.30E-08 | 19.23 | 0.46 | 0.00 |
| 3-hydroxypropyric acid | 0.79 | 4.85 | 1.29 | 3.06E-59 | 3.74 | 1.32 | 6.79E-29 |
| 3-hydroxybenzene | 1.89 | 4.64 | 0.50 | 2.79E-19 | 3.48 | 0.55 | 5.03E-08 |
| 3,6-epoxy-decane | 2.17 | 9.99 | 1.17 | 2.21E-51 | 8.58 | 1.22 | 8.96E-38 |
| 2-deoxy-4 hydroxyacetyl acetic acid | 0.69 | 9.82 | 1.47 | 4.26E-60 | 7.05 | 1.44 | 3.26E-31 |
| 2-ketone-isohexic acid | 0.56 | 4.95 | 1.48 | 5.88E-89 | 3.31 | 1.36 | 2.06E-51 |
| 2-ketone diacinate | 0.71 | 12.15 | 1.49 | 7.83E-31 | 7.08 | 1.34 | 5.73E-17 |
| 2-oxydyric acid | 0.88 | 3.93 | 0.74 | 8.26E-47 | 3.32 | 0.96 | 1.41E-14 |
| 2-hydroxyydrine | 0.45 | 3.38 | 0.94 | 9.14E-49 | 2.35 | 1.06 | 3.74E-37 |
| 2-hydroxymauric acid | 1.07 | 5.32 | 0.85 | 1.41E-32 | 3.94 | 0.91 | 4.60E-12 |
| 2-hydroxyfloweric acid | 1.04 | 5.39 | 1.37 | 1.57E-74 | 3.75 | 1.41 | 7.11E-50 |
| 2-hydroxyethysedric acid | 0.67 | 4.64 | 1.43 | 1.07E-78 | 3.20 | 1.35 | 1.24E-43 |
| Utou acid | 21.33 | 53.76 | 0.75 | 1.44E-32 | 36.30 | 0.51 | 3.48E-09 |

Results of the comparison of urine organic acids values are the peak area ratio of the corresponding index.

**Supplementary Table 2**

**PCA model parameter table**

| **Group** | **Type** | **A** | **N** | **R2X (cum)** |
| --- | --- | --- | --- | --- |
| GDD | PCA | 3 | 963 | 0.603 |
| ID | PCA | 3 | 467 | 0.617 |

A: The number of principal components of the model.

N: The number of observations of the model (here is the number of samples).

R2X (cum): Represents the cumulative explanatory degree of the model to the X variable.

**Supplementary Table 3**

**OPLS-DA model parameter table**

| Group | Type | A | N | R2X (cum) | R2Y(cum) | Q2 (cum) |
| --- | --- | --- | --- | --- | --- | --- |
| GDD | OPLS-DA | 3 | 963 | 0.539 | 0.808 | 0.802 |
| ID | OPLS-DA | 3 | 467 | 0.515 | 0.655 | 0.638 |

A: The number of principal components of the model.

N: The number of observations of the model (here is the number of samples).

R2X (cum): Represents the cumulative explanatory degree of the model to the X variable.

R2Y (cum): Represents the cumulative explanatory degree of the model to the Y variable.

Q2 (cum): The predictability of the model.
